# Novel Validated Analytical Method Based on Potentiometric Transduction for the Determination of Citicoline Psychostimulant/Nootropic Agent

**DOI:** 10.3390/molecules25153512

**Published:** 2020-07-31

**Authors:** Ayman H. Kamel, Abd El-Galil E. Amr, Hoda R. Galal, Abdulrahman A. Almehizia

**Affiliations:** 1 Department of Chemistry, Faculty of Science, Ain Shams University, Cairo 11566, Egypt; 2 Pharmaceutical Chemistry Department, Drug Exploration & Development Chair (DEDC), College of Pharmacy, King Saud University, Riyadh 11451, Saudi Arabia; mehizia@ksu.edu.sa; 3 Applied Organic Chemistry Department, National Research Center, Dokki 12622, Giza, Egypt; 4 Inorganic Chemistry Department, National Research Center, Dokki 12622, Giza, Egypt; hrgalal@hotmail.com

**Keywords:** citicoline, psychostimulant/nootropica, potentiometric sensors, method validation, pharmaceutical formulations

## Abstract

Herein, a novel validated potentiometric method is presented for the first time for citicoline determination. The method is based on measuring the potential using new constructed citicoline electrodes. The electrodes are based on the use of citicolinium/phosphomolybdate [Cit]_2_[PM] (sensor I) and citicolinium/tetraphenylborate [Cit][TPB] (sensor II) ion association complexes. These sensory materials were dispersed in plasticized polyvinyl chloride (PVC) polymeric membranes. The sensors revealed a Nernstian response with the slopes 55.9 ± 1.8(*r^2^* = 0.9994) and 51.8 ± 0.9 (*r^2^* = 0.9991) mV/decade over a linearity range of 6.3 × 10^−6^–1.0 × 10^−3^ and 1.0 × 10^−5^–1.0 × 10^−3^ M and detection limits of 3.16 × 10^−6^ and 7.1 × 10^−6^ M for sensors I and II, respectively. To ensure the existence of monovalent citicoline, all measurements were performed in 50 mM acetate buffer at pH 3.5. All presented electrodes showed good performance characteristics such as rapid response, good selectivity, high potential-stability and long life-span. Method verification and validation in terms of response linearity, quantification limit, accuracy, bias, trueness, robustness, within-day variability and between-days variability were evaluated. The method was introduced for citicoline determination in different pharmaceutical formulations and compared with the standard high performance liquid chromatography (HPLC) method.

## 1. Introduction

**Figure 1:**
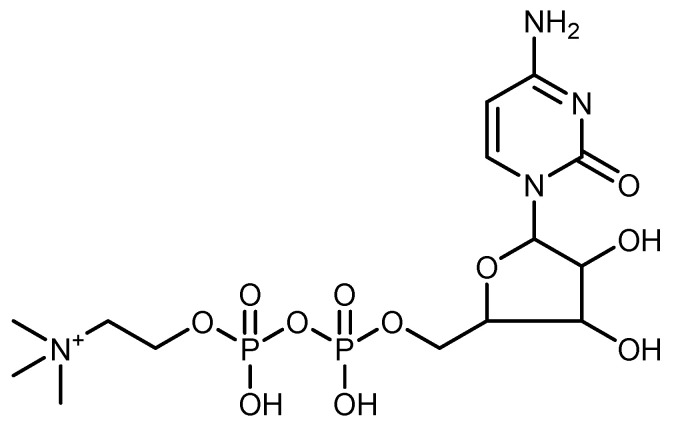
Chemical structure of citicoline [cytidine 5-diphosphocholine].

Citicoline (CIT, cytidine 5′-trihydrogen diphosphate, Figure 1) is considered a psychostimulant/ nootropic agent. It has been used as a medicine in treating cerebral insufficiency and associated neurological disorders such as strokes, brain traumas, and Parkinsonism [1]. It also works to repair damaged cholinergic neurons by increasing acetylcholine production and reduces the accumulation of fatty acids at the site of damaged nerves caused by stroke [2,3]. Different analytical techniques were reported in the literature for citicoline determination in different pharmaceutical formulations. These techniques include spectrophotometry [4,5,6,7,8,9,10,11], spectrofluorimetry [12], densitometry [13], high performance liquid chromatography [14,15,16,17,18,19,20,21,22,23,24]. All these methods suffer from different severe limitations such as expensive instruments being required, being time consuming, non reliable results being obtained, multiple steps for sample preparation being needed and unstable reagent being used.

Electrochemical sensors based on potentiometric transduction are characterized by many distinctive advantages. They are characterized by their high sensitivity of measurement, good selectivity, easy operation and they can be integrated in automatic systems. They have been widely used successfully in detecting several analytes in different fields [25,26,27,28,29,30]. Polymeric membrane ion-selective electrodes (ISEs) turned out to be reliable potentiometric-sensing devices with special advantages, including simplicity and ease of construction, the ability to be miniaturized, low cost of production, and mechanical stability. ISEs are good from a practical point of view because they can have various shapes and sizes, as well as being work in any position [31,32,33,34]. Till now no electrochemical sensor based on potentiometric determination is reported for citicolin determination.

In this work, cost-effective, robust and reliable potentiometric sensors were prepared, characterized and presented for citicoline detection in different pharmaceutical formulations. The sensors are based on the ion association citicoline/tetraphenylborate ([CIT][TPB]) and citicoline/phosphomolybdate ([CIT]_2_[HPMo_12_O_40_]) complexes in a plasticized polyvinyl chloride (PVC matrix). The performance potentiometric characteristics of the suggested electrodes such as linearity, sensitivity, selectivity, and reliability were studied. The sensors were used for free citicoline detection in different pharmaceutical formulations.

## 2. Results and Discussions

### 2.1. Response Characteristics of the Sensors

**Figure 2:**
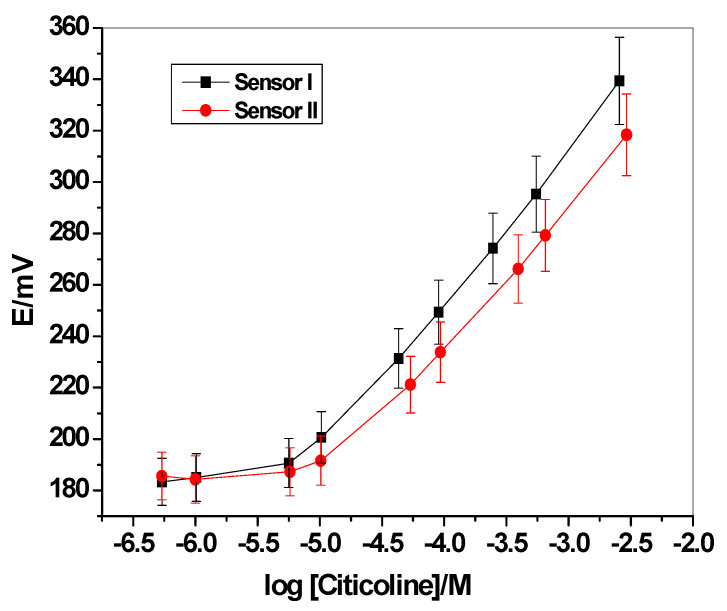
Calibration plot of the suggested citicoline membrane-based sensors.

Citicoline reacted with both phosphomolybdic acid and sodium tetraphenylborate at pH 3.5, forming citicolinium/phosphomolybdate [Cit]_2_[PM] (sensor I) and citicolinium/tetraphenylborate [Cit][TPB] (sensor II) ion association complexes in the ratios 2:1 and 1:1, respectively. These sensory materials were dispersed in a plasticized PVC matrix. According to IUPAC recommendations [35], the performance response characteristics of the presented sensors were evaluated and presented in Table 1. Calibration plot of the sensors were presented in Figure 2.

The time response of the proposed electrodes can be calculated after recording the time taken to reach 95% of the steady-state potential. A steady-state potential with ±0.3 mV was achieved by both electrodes after their immersion by a 10-fold increase of citicoline concentration starting from 10^−6^ to 10^−3^ M. The response time was observed to be less than 10 s for all citicoline concentrations. This confirms the fast response of these sensors and the rapid determination of citicoline.

Several calibrations for each sensor were performed to check the long-term potential stability. It was observed that the electrodes revealed a low-potential drift, and long-term stability. For all examined electrodes, the detection limits, linear range and calibration slopes were reproducible within ±2.3% of their original values over a period of at least 8 weeks.

### 2.2. Method Validation

Method validation for the presented protocol was performed using the quality control/quality assurance standards and guidelines [36,37]. All performance characteristics of the method such as linearity range, detection limit, accuracy, trueness, bias, within-day repeatability, between-days reproducibility, robustness, selectivity and uncertainty were evaluated to verify the suitability of the presented method for citicoline assessment.

#### 2.2.1. Method Linearity and Quantification Limit

Within the citicoline concentration range (1.0−1000µM), the linearity of the proposed method was evaluated using six batches (six determinations each) covering the previously mentioned citicoline concentration range. The sensors displayed a linear dynamic range of 6.3 × 10^−6^–1.0 × 10^−3^ and 1.0 × 10^−5^–1.0 × 10^−3^ M 55.9 ± 1.8 (*r^2^* = 0.9994) and 51.8 ± 0.9 (*r^2^* = 0.9991) mV/decade for sensors I and II, respectively.

The quantification limit was calculated from the intersection of the extrapolated linear segment of the constructed calibration plot. The sensors revealed a limit of detection 3.16 × 10^−6^ and 7.1 × 10^−6^ M for sensors I and II, respectively.

#### 2.2.2. Repeatability and Reproducibility

The spread of results obtained for citicoline concentration (i.e., 10 µg/mL) when measured within the same day and in different days under different conditions and different sensor assembly is also evaluated. Reproducibility (*R*) is obtained from the standard deviation (*S_R_*) calculated by:

*R* = 2.8 × *S_R_* (1)

The data reproducibility within-day was found to be 0.9% and 1.1% while between-day variability was 1.2% and 1.5% for sensors I and II, respectively.

#### 2.2.3. Data Precision and Data Accuracy

Method precision was calculated from the standard deviation (*S*) and the average results (*X*) (*n* = 6) of citicoline reference standard sample (10 µM) using the equation:

*Precision*, % = (*S*/*X*) × *100* (2)

Replicate measurements of internal quality control citicoline samples containing 10.0, 50.0 and 100 µM citicoline (*n* *= 6*, each) presented relative standard deviations (*RSD*, %) in the range of 1.1± 0.1 and 1.2 ± 0.2%, for sensors I and II, respectively. The absolute uncertainty is expressed as: *X* citicoline value ± precision.

Method accuracy was assessed through spiking a known concentration of citicoline (0.2 µM) to a reference sample (10 µM). The degree of data closeness is a measure for the accuracy. The method accuracy within the calculated linear range of the presented analytical protocol was calculated and found to be 98.1 ± 0.7 and 97.3 ± 1.1% for sensors I and II, respectively. Accuracy was calculated using the equation:

Accuracy, % = [(*X_s_*-*X*)/*X_add_*] × *100* (3)

where *X_s_* is the mean of results obtained for measuring the spiked citicoline solution, *X* is the mean of results obtained for the un-spiked citicoline reference samples, and *X_add_* is the amount of added citicoline standard.

#### 2.2.4. Method Robustness

Method robustness refers to the ability of the presented method to resist any sudden change in method parameters. The pH effect on the electrode behavior was examined by immersing either sensor I or II in conjunction with a pH glass electrode and a reference electrode in 10^−4^ and 10^−3^ M citicoline solutions. The pH of the solutions was adjusted over the pH range 2−11 using either NaOH or HCl solutions. It was observed that no remarkable change took place in the response of the electrodes within the range 3−4.5. Within this range, citicoline exists in its cationic form. Above this range, the response declined due to the existence of the un-sensed neutral citicoline. At pH above 8.5, a sharp decrease in the potential response was recorded due to the formation of citicoline–sodium in its anionic form. So, all measurements were carried out in 50 mM acetate buffer at pH 3.5. This is to ensure the presence of monovalent citicoline in its cationic form. Sodium Chloride (30 mM) was used with the buffer to fix the ionic strength of the solution.

#### 2.2.5. Method Selectivity

Selectivity of the developed method toward citicoline was checked over many other interfering ions. The selectivity coefficient (*K^pot^_Cit_^+^_,j_*) values were calculated after applying the so called “modified separate solution method (MSSM)” for selectivity evaluation [38]. As shown in Table 2, sensor I showed higher selectivity behavior towards citicoline over choline, caffeine, glutamine, cysteine, urea, aminomethane, dimethylamine, quinine, Ba^2+^, Ca^2^^+^, and Mg^2^^+^ ions than sensor II. On the other hand, sensor II exhibited better selectivity than sensor I over K^+^, histidine, 1,2-diaminoethane, hexamine, hydroxylamine, ephedrine, codeine, morphine, and alanine.

### 2.3. Analytical Applications

Fortamind ampoule and Somazina oral drop are pharmaceutical formulations containing citicoline and were collected from the local market. The content of citicoline in these formulations was investigated using the proposed validated method as presented. The data obtained showed an acceptable average recovery of 98.6 and 99.4 with a mean standard deviation of ±2.2% and ±1.9% for sensors I and II, respectively. The results were compared with that obtained from the reported HPLC method in US pharmacopeia [39]. The t-Student and F-tests revealed no significant difference between the set of data obtained by the two methods. This confirms the successful applicability of the presented validated method and its efficiency for citicoline determination in different pharmaceutical formulations. All results are shown in Table 3.

## 3. Materials and Methods

### 3.1. Apparatus

A digital pH/mV meter (Orion SA 720, Boston, MA, USA) was used for potential measurements at room temperature. The electrochemical cell is composed from the proposed citicoline electrodes in conjunction with an Ag/AgCl double junction reference electrode, in which its outer compartment was filled with 0.1 M CH_3_COOLi solution. A Ross combined glass pH electrode was used for all pH solution adjustment.

### 3.2. Chemicals and Reagents

All chemicals used in this work were of analytical reagent grade and used without any further purification. Phospholybdic acid (H_3_PMo_12_O_40_), high molecular weight polyvinyl chloride (PVC), 2-nitrophenyl octyl ether (*o*-NPOE), tetrhydrofuran (THF) and sedum tetraphenyl borate (Na-TPB) were purchased from Sigma-Aldrich Chemical Co. (St. Louis, MO, USA). Citicoline (99.50% purity) was kindly supplied as a gift from Amriya Pharmaceutical Industries (Cairo, Egypt).

Stock citicholine solution (10^−2^ M) was prepared in 50 mM acetate buffer (pH 3.5) and 30 mM NaCl. Working citicoline solutions (10^−6^−10^−3^ M) were prepared by accurate dilutions using 50 mM acetate buffer, pH 3.5.

### 3.3. Sensor Preparation and Potential Measurements

Citicholinum phosphomolybdate (Cit/PMA) (sensor I) and citicolinium tetraphenylborate (Cit/TPB) (sensor II) ion association complexes were synthesized after mixing equal volumes of 10^−2^ M citicoline (pH 3.5) with 10^−1^ M of either phosphomolybdic acid (PMA) or sodium tetraphenylporate (Na-TPB), respectively. The precipitates formed were filtered, washed and then dried. The membrane-based sensors were prepared after dissolution of 3 mg of each ion-associate with 65 mg PVC and 124 mg *o*-NPOE as a plasticizer in 3 mL THF. The mixture was placed in a 3.3 cm Petri-dish and left overnight for complete solvent evaporation. The resulting polymeric membrane was peeled off and 9 mm i.d discs were glued onto a 7 mm i.d PVC Tygon tube using THF. A 10^−3^ M citicolin (pH 3.5) was used as an inner filling solution, and a 3 mm diameter Ag wire coated with AgCl layer was used as an internal reference electrode. The membrane sensors were soaked for conditioning in 10^−3^ M citicoline solution overnight and were stored in distilled water when not in use.

Calibration of the proposed sensors was done after their immersion with the reference electrode into a 25 mL beaker containing 9 mL of 50 mM acetate buffer solution of pH 3.5. Aliquots (0.5−1.0 mL) of 10^−6^−10^−3^ M standard citicolinium solutions were successively added followed by measuring of the potential response after each subsequent addition. A calibration plot was constructed by plotting the potential reading versus log (citicoline) concentrations. The calibration-plot was used for all subsequent determination of unknown citicoline samples.

### 3.4. Citicoline Assessment

Different pharmaceutical formulations containing citicoline were collected from the local market to assess citicoline content using the proposed sensors. These formulations include Fortamind ampoule (500 mg/4 mL, Globe international pharmaceuticals, Cairo, Egypt) and Somazine oral drop solution (500 mg/4 mL, October pharma, 6th October City, Egypt). Aliquots (0.5−1.0 mL) from each formulation sample was diluted in a 50 mL measuring flask and then completed with 50 mM acetate buffer solution at pH 3.5 to the mark. The potential reading of each sample solution was recorded and compared with the calibration plot for the standard citicoline solutions.

## 4. Conclusions

A novel, facile and validated method based on potentiometric transduction was presented for the first time to quantify trace amounts of citicoline psychostimulant/nootropic agent. The method is based on new constructed and characterized liquid-contact electrodes. The sensory materials used in the polymeric electrode membrane were the ion association of citicoline with phosphomlybdic acid (PMA) ([Cit]_2_[PM]) and sodium tertaphenyl borate (Na-TPB) ([Cit][TPB]). These complexes were dispersed in a plasticized PVC polymeric matrix. The sensors exhibited a Nernstian slopes over wide linear ranges and good detection limits as presented in Table 1. The measurements were carried out in 50 mM acetate buffer of pH 3.5. Validation of the presented protocol with the suggested electrodes, by calculating the detection limit, sensitivity, accuracy, precision, bias, within-day-variability and between-day-variability exhibited good performance characteristics. As compared to the previously reported methods for citicoline detection, the presented potentiometric method overcomes the limitations shown by these methods. It offered low-cost instruments for use, low time of analysis is taken, reliable results are obtained, no sample preparation is needed, and unstable reagents are not used. This confirms the successful applicability for rapid and accurate determination of citicoline in different pharmaceutical formulations collected from the local market.

## Figures and Tables

**Table 1:** Performance characteristics of citicoline membrane-based electrodes in 50 mM acetate buffer, pH 3.5.

Parameter	Sensor I	Sensor II
**Slope ^a^ mV/decade**	55.9 ± 1.8	51.8 ± 0.9
**Correlation coefficient (r^2^)**	0.9994	0.9991
**Linear range, M**	6.3 × 10^−6^−1.0 × 10^−3^	1.0 × 10^−5^−1.0 × 10^−3^
**Detection limit, M**	3.16 × 10^−6^	7.1 × 10^−6^
**Working range, pH**	3.0−4.5	3.0−4.5
**Response time, s**	<10	<10
**Lifespan, week**	8	8
**Accuracy, %**	99.3	99.1
**Within-day variability Cv_w_ (%)**	0.9	1.1
**Between-day variability Cv_b_ (%)**	1.2	1.5

^a^ Average of 5 measurements.

**Table 2:** Selectivity coefficients (*K^pot^_Cit_^+^_,j_*) of citicoline membrane based sensors.

Interfering Ion, *I*	* Log *K^pot^_Cit_^+^_,j_*	
	Sensor I	Sensor II
Choline	−2.61 ± 0.3	−1.74 ± 0.2
Codeine	−1.76 ± 0.2	−2.95 ± 0.3
Morphine	−1.80 ± 0.4	−2.78 ± 0.4
Ephedrine	−1.67 ± 0.4	−2.40 ± 0.2
Caffeine	−4.57 ± 0.2	−4.29 ± 0.6
Histidine	−4.03 ± 0.2	−4.85 ± 0.5
Glutamine	−3.85 ± 0.1	−3.54 ± 0.4
Quinine	−3.47 ± 0.2	−3.34 ± 0.3
Cysteine	−4.62 ± 0.3	−1.55 ± 0.2
Aminomethane	−3.32 ± 0.5	−3.22 ± 0.6
Dimethylamine	−3.45 ± 0.2	−3.31 ± 0.5
1,2-diaminoethane	−4.53 ± 0.5	−4.64 ± 0.2
Alanine	−4.12 ± 0.3	−4.56 ± 0.5
Hydroxylamine	−4.21 ± 0.3	−4.42 ± 0.1
Hexamine	−3.55 ± 0.4	−3.65 ± 0.2
Urea	−4.98 ± 0.2	−4.76 ± 0.1
Mg^2+^	−5.96 ± 0.3	−5.70 ± 0.2
Ca^2+^	−5.75 ± 0.4	−5.32 ± 0.3
Ba^2+^	−5.12 ± 0.7	−5.01 ± 0.6
K^+^	−5.01 ± 0.5	−5.11 ± 0.7

* Average of 3 measurements.

**Table 3:** Assessment of citicoline in different pharmaceutical formulations using the presented electrodes.

Product and Its Source	Nominal Content, mg/4 mL	Found Content, mg/4 mL				^b^*t*-Test	^b^*F*-Test
		Proposed Method	^a^ Recovery %± SD	Reference Method [39]	^a^ Recovery %± SD		
Fortamind ampoule (Globe international pharmaceuticals, Cairo, Egypt)	500	496.4 ± 2.1	99.3 ± 0.3	498.7 ± 1.1	99.7 ± 0.2	1.2	1.1
Somazine oral drop solution (October pharma 6 October City, Egypt)	500	491.6 ± 1.3	98.3 ± 0.2	496.3 ± 0.6	99.2 ± 0.1	2.2	2.1

^a^ Mean of three replicate measurements ± standard deviation (SD). ^b^ t-Student and *F* test at 95% confidence level are 4.30, 19.00 respectively.
